# Screening self-peptides for recognition by mouse alloreactive CD8^+^ T cells using direct ex vivo multimer staining

**DOI:** 10.1016/j.xpro.2022.101943

**Published:** 2022-12-15

**Authors:** Eric T. Son, Moumita Paul-Heng, Mario Leong, Chuanmin Wang, Alexandra E. Hill, Martina Denkova, Anthony W. Purcell, Nicole A. Mifsud, Alexandra F. Sharland

**Affiliations:** 1 Transplantation Immunobiology Group, University of Sydney Central Clinical School, Charles Perkins Centre, Faculty of Medicine and Health, Sydney, NSW 2006, Australia; 2 Infection and Immunity Program, Department of Biochemistry and Molecular Biology, Biomedicine Discovery Institute, Monash University, Clayton, VIC 3800, Australia

**Keywords:** Cell Biology, Cell isolation, Immunology, Model Organisms, Molecular Biology, Antibody

## Abstract

Here, we present a protocol to identify immunogenic self-peptide/allogeneic major histocompatibility complex (MHC) epitopes. We describe the generation of enriched alloreactive CD8+ T cells by priming mice with a skin graft expressing the allogeneic MHC class I molecule of interest, followed by boosting with a liver-specific AAV vector encoding the heavy chain of that donor MHC allomorph. We then use a peptide-exchange approach to assemble a range of peptide-MHC (pMHC) multimers for measuring recognition of the various epitopes by these alloreactive T cells.

For complete details on the use and execution of this protocol, please refer to Son et al. (2021).^1^

## Highlights

• In vivo generation of alloreactive T cells
• Peptide-exchange approaches allow rapid generation of many pMHC multimer specificities
• Can be adapted for screening of tissue-specific peptides
• Downstream applications include scRNA-seq and TCR sequencing

## Abstract (editor highlights)

Publisher’s note: Undertaking any experimental protocol requires adherence to local institutional guidelines for laboratory safety and ethics.

## Graphical abstract

**Figure:**
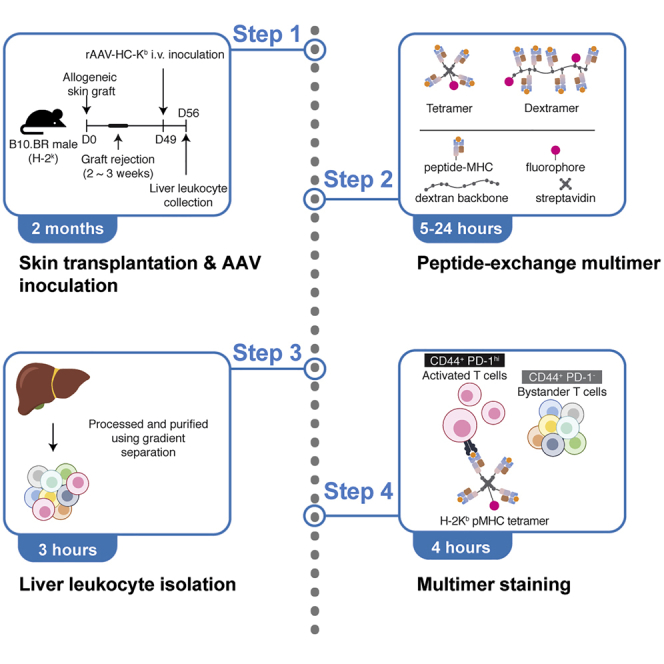

## Before you begin

The protocol below describes the specific steps for identification of immunogenic self-peptides presented by H-2K^b^. For B10.BR recipients, we used 178.3 mice (K^b^ transgenic on a B10.BR background) as skin donors while for BALB/c recipients, the skin donors were C57BL/6 (H-2^b^) mice. We have also used this protocol to identify immunogenic peptides bound by H-2K^d^. Here, we used isolated liver leukocytes after a prime-boost as the source of a highly enriched alloreactive population. Alternatively, infiltrating T cells from transplanted organs (e.g., heart, kidney) may be used if examination of tissue-specific peptides is desired. QuickSwitch Custom Tetramer Kits (MBL International) were utilized to generate multiple tetramers in order to screen an array of >100 pMHC epitopes. Several other commercially-available reagents enable peptide MHC exchange or loading (BioLegend Flex-T – UV-mediated exchange monomer, ImmunAware easYmer, TetramerShop Empty Loadable Tetramer, etc). In a preliminary evaluation, comparable results were obtained with different peptide-exchange methods (Figure 6). Chaperone-mediated peptide exchange is another recently-reported method that would be suitable for use with this protocol.^2^

### Institutional permissions

All animal procedures were approved by the University of Sydney Animal Ethics Committee (protocols 2017/1253 and 2022/2092) and carried out in accordance with the Australian code for the care and use of animals for scientific purposes. Biosafety approval for AAV vectors was granted by the University of Sydney Institutional Biosafety Committee (IBC approval number 17NO28 and 22N012).

### AAV vector preparation

The AAV2/8 vector encodes murine MHC class I heavy chain (H-2K^b^) under the liver-specific human α-1 antitrypsin promoter and human ApoE enhancer, flanked by AAV2 inverted terminal repeats. Construct sequences for H-2K^b^ and H-2K^d^ vectors are available.^3^ The AAV vector was packaged, purified, and quantitated by the Vector and Genome Engineering Facility, Children’s Medical Research Institute, Westmead, Australia.^4^

### Peptide selection

In our example, a total of 100 peptides were selected for screening.^1^ Choose peptides for screening based on the allomorph and tissues of interest. Details of the immunopeptidome for the desired tissue/MHC combination may be obtained from publicly-available datasets^1^^,^^5^ or may be determined empirically.^1^^,^^6^

## Key resources table

**Table:** 

REAGENT or RESOURCE	SOURCE	IDENTIFIER
**Antibodies**		

Mouse TruStain FcX (1 μg in 100 μL)	BioLegend	Cat#101320
Anti CD8a FITC / clone : KT15 (0.25 μg in 100 μL)	Bio-Rad	Cat#MCA609FB
^&^Anti CD90.2 PerCPCy5.5 / clone : 53-2.1 (0.25 μg in 100 μL)	BioLegend	Cat#140322
Anti CD44 APC / clone : IM7 (0.5 μg in 100 μL)	BioLegend	Cat#103012
Anti PD-1 BV421 / clone : 29F.1A12 (1 μg in 100 μL)	BioLegend	Cat#135218
Anti CD19 PECy7 / clone : 6D5 (0.25 μg in 100 μL)	BioLegend	Cat#115520
Anti CD14 PECy7 / clone : Sa14-2 (1 μg in 100 μL)	BioLegend	Cat#123316
Anti PE purified / clone : PE001 (0.5 μg in 100 μL)	BioLegend	Cat#408101
Zombie NIR (viability dye)	BioLegend	Cat#423105

**Bacterial and virus strains**		

AAV2/8 encoding murine MHC class I heavy chain (H-2 K^b^)	Vector and Genome Engineering Facility	Custom order

**Chemicals, peptides, and recombinant proteins**		

Dasatinib	Sigma-Aldrich	Cat#CDS023389
D-Biotin	Sigma-Aldrich	Cat#711610
Sodium azide	Sigma-Aldrich	Cat#S2002-5G
Dimethyl sulfoxide (DMSO)	Sigma-Aldrich	Cat#D8418-50ML
Phosphate buffered saline (PBS)	Lonza	Cat#BE17-516F
RPMI-1640 medium supplemented with L-glutamine	Lonza	Cat#12-702F
Fetal calf serum (FCS)	Sigma-Aldrich	Cat#13K179
Isotonic Percoll™ PLUS	Cytiva	Cat#17-5445-01
Synthetic peptides	GL Biochem Shanghai Ltd	Custom order
PE Streptavidin	BioLegend	Cat#405203
∗QuickSwitch™ Quant H-2 Kb Tetramer Kit-PE	MBL International	Cat#TB-7400-K1
∗BioLegend Flex-T™ H-2Kb Monomer UVX	BioLegend	Custom order
∗Tetramer Shop Empty Loadable Tetramer	Tetramer Shop	MKb-016
∗ImmunAware H2-Kb easYmer® kit	ImmunAware	5004-01
Custom MHC Dextramer™, H-2 Kb / SNYLFTKL / PE	Immudex	Cat#JD5714-PE
Custom MHC Dextramer™, H-2 Kb / VGPRYTNL / PE	Immudex	Cat#JD5905-PE
Custom MHC Dextramer™, H-2 Kb / RTYTYEKL / PE	Immudex	Cat#JD5907-PE
Klickmer®-PE	Immudex	Cat# DX01K PE 200

**Critical commercial assays**		

#QuickSwitch™ Quant H-2 Kb Tetramer Kit-PE (Kit includes quantitation reagents)	MBL International	Cat#TB-7400-K1
#LEGEND MAX™ Flex-T™ Human Class I Peptide Exchange ELISA Kit (NB: detects human beta-2-microglobulin which is also used in folding H-2 monomers)	BioLegend	Cat#447207

**Deposited data**		

scRNA-seq data regarding dasatinib treatment	Paul-Heng et al. (unpublished)	NCBI GEO: GSE217149

**Experimental models: Organisms/strains**		

Mouse: B10.BR (male, 8–12 weeks)	Laboratory Animal Services (The University of Sydney)	N/A
Mouse: 178.3 (male, 8–12 weeks)	Laboratory Animal Services (The University of Sydney)	N/A

**Software and algorithms**		

FlowJo version 10	Becton Dickinson	https://www.flowjo.com/solutions/flowjo/downloads
GraphPad Prism version 8.01	GraphPad Prism version GraphPad Software	https://www.graphpad.com/updates/prism-801-release-notes
Seurat4.0	Satija Lab	https://cran.r-project.org/web/packages/Seurat/index.html
WTA Analysis Pipeline (Revision 7)	BD Biosciences	https://scomix.bd.com/hc/en-us/articles/360047408451-BD-Rhapsody-Analysis-Pipeline-Updates

**Other**		

Isoflurane	Baxter	Cat#AHN3637
Cyanoacrylate adhesive	Dermabond	Cat#ANX12
Chlorhexidine 0.5% in 70% Alcohol solution	Pfizer	Cat#PC-16062503
Buprenorphine	Temvet	N/A
Ampicillin	Alphapharm	N/A
Sodium chloride 0.9%	Pfizer	Cat#PHA19042010-P
Syringe 1 mL Luer Slip Tuberculin BD	BD	Cat#BD302100
Syringe 20 mL Luer Lock BD	BD	Cat#BD300141
30G 1/2 (0.3 mm × 13 mm) BD Microlance Sterile Needles	BD	Cat#BD304000
SURFLO IV Catheters 22G × 25 mm Blue	Terumo	Cat#TE-OX2225C
Surgical Scalpel Blade No. 21	Swann-Morton	N/A
Surgical forceps and scissors	(Various sources available)	N/A
STERI250 Glass dry bead sterilizer	Able Scientific	N/A
Physitemp TCAT-2DF Controller	Physitemp Instruments	N/A
Heating pad	Wombaroo Passwell	N/A
Handheld UV Lamp, LW/SW, 6 W, UVGL-58	Scientifix	N/A
Fortessa X-20 Cytometer	BD Biosciences	N/A

***Note:*** ∗and # are alternatives.

***Note:***^&^CD90 was used to as an alternative to CD3 in identification of T cells to avoid gating out activated T cells that had downregulated CD3. The isoform of CD90 in B10.BR, BALB/c and C57BL/6 mice is CD90.2.

## Step-by-step method details

### Part 1: Mouse skin transplantation

**Timing: 30 min per skin graft**

Full-thickness skin grafts can be obtained from back^7^ or tail skin. Here we describe a method for tail skin grafts.

**Figure 1:**
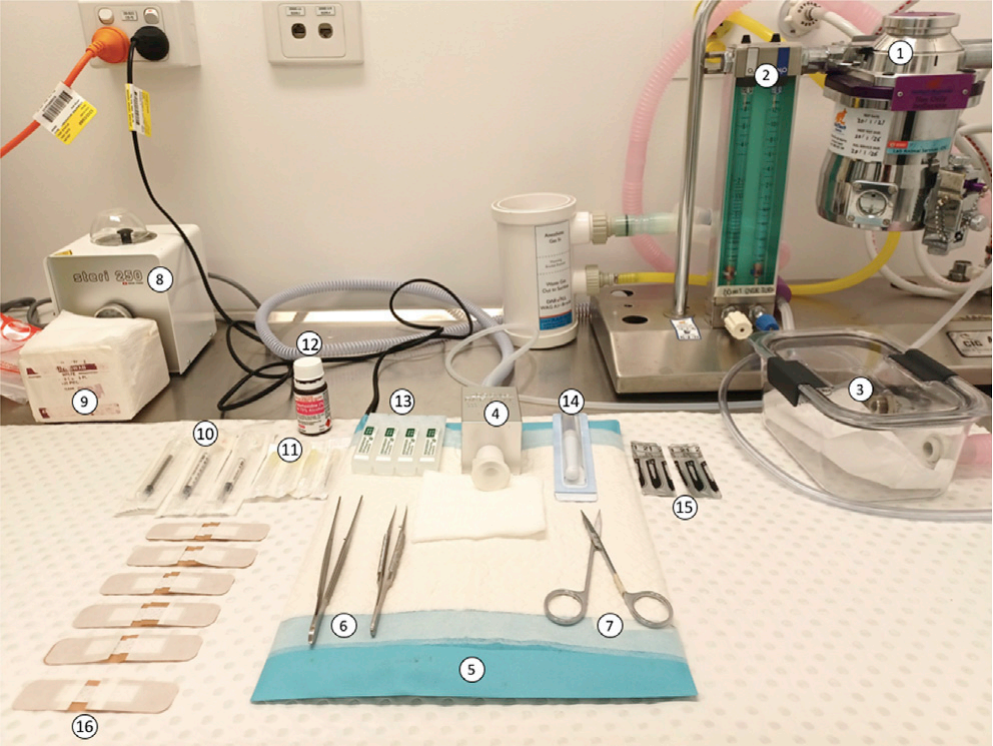
Skin transplantation surgery materials Components are 1: isoflurane mouse anaesthetic machine, 2: flow regulator, 3: anaesthetic induction chamber, 4: nose-cone, 5: warming pad, 6: surgical forceps, 7: surgical scissors, 8: bead steriliser, 9: sterile gauze, 10: syringes for saline, ampicillin and buprenorphine injections, 11: 30 gauge needles, 12: Chlorhexidine 0.5% in 70% ethanol solution, 13: sterile normal saline, 14: Dermabond surgical adhesive, 15: size 21 curved scalpel blade, 16: non-adhesive absorptive dressing.

Assemble the materials as shown in Figure 1.

#### Donor skin collection

**Timing: 15 min per donor mouse**

**Figure 2:**
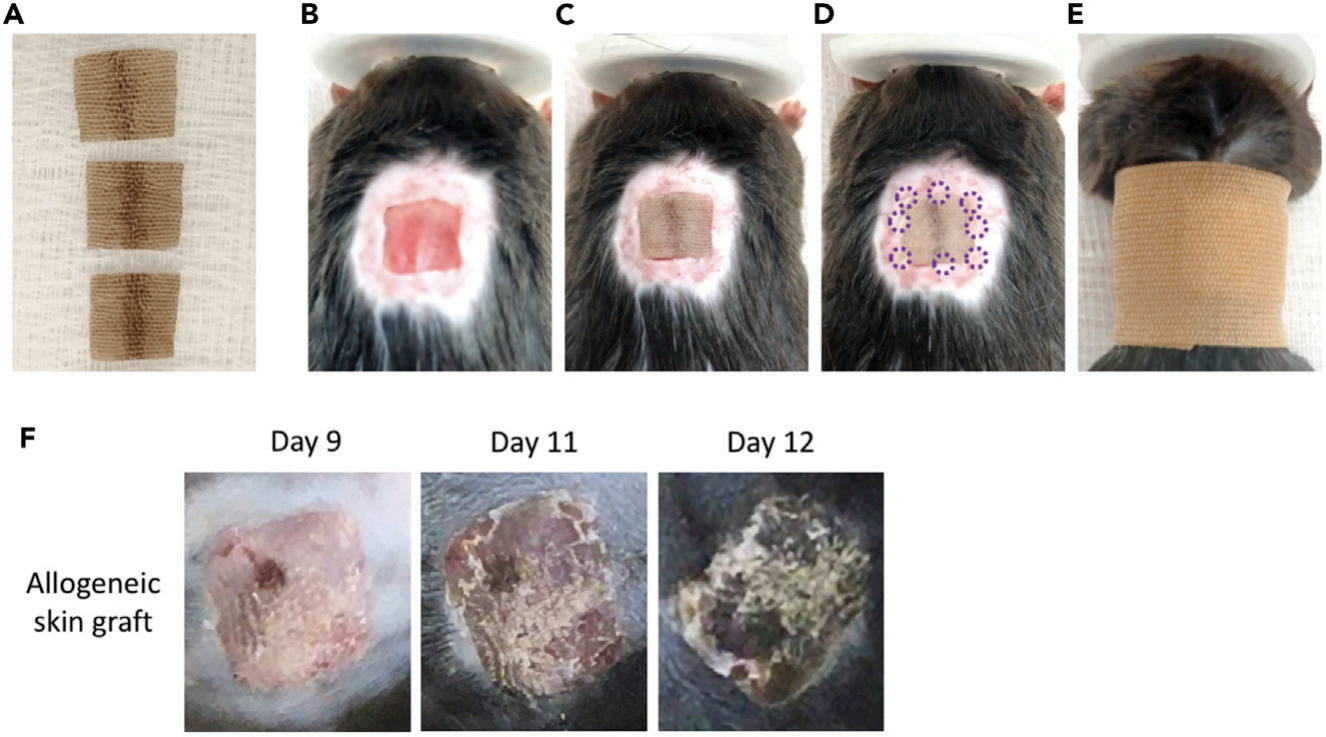
Skin transplantation procedure and monitoring (A) Representative macroscopic images showing grafts prepared from a donor tail ready to transplant. (B–E) Representative macroscopic images showing the skin transplantation procedure for recipient mice. Left to right: (B) prepared graft bed, (C) graft immediately after placement, (D) graft after tissue adhesive applied, (E) bandaged mouse. (F) Representative macroscopic images demonstrating the process of graft rejection. The first image (Day 9) shows a well healed-in skin graft at 9 days post-transplant. The middle image (Day 11) shows an inflamed skin graft undergoing rejection. The last image (Day 12) shows a necrotic skin graft on the day of rejection by the recipient mouse.

1. Autoclave surgical instruments at the beginning of each daily session of surgery.
2. Weigh donor mice pre-operatively and record their weights.
3. Induce anaesthesia by using isoflurane at 3% and supplemental oxygen at 0.6 L/min.
4. Check the tail pinch reflex to ensure sufficient depth of anaesthesia before an incision is made.
5. Amputate the donor tail using a scalpel and euthanise the donor mouse by isoflurane overdose with cervical dislocation under anaesthesia.
6. Collect the tail skin by making a cranial to caudal inferior midline incision in the amputated tail.
7. Mobilise the tail skin and remove it from the musculoskeletal structures.
8. Irrigate the tail skin with sterile normal saline containing ampicillin (10 mg/mL).
9. Flatten the tail skin and cut it into squares measuring approximately 10 mm × 10 mm.
10. Store in sterile saline/ampicillin soaked gauze over ice in preparation for grafting (Figure 2A).

#### Recipient skin transplantation

**Timing: 15 min per recipient mouse**

11. Weigh recipient mice pre-operatively and record their weights.
12. Induce anaesthesia with isoflurane at 3% and supplemental oxygen at 0.6 L/min.
13. Place the recipient mouse on a heat pad, separated from the pad by 2 layers of sterile gauze.
14. Inject the recipient mouse subcutaneously with buprenorphine (0.05 mg/kg, 50 μL) for analgesia, prophylactic ampicillin (100 mcg/kg, 100 μL) and 1 mL of preheated (41°C) sterile normal saline (divided between 2 locations).
15. Apply a lubricating eye ointment to both eyes and insert a lubricated rectal probe for temperature monitoring.
16. Move the recipient mouse to the ventral position and prepare the dorsal skin with chlorhexidine 0.5% in 70% alcohol solution.
17. Shave the back and lateral thoracic regions with a scalpel blade from the cervical to the distal thoracic levels.
18. Check the tail pinch reflex to ensure sufficient depth of anaesthesia before an incision is made.
19. Prepare a graft bed on the back by sharp dissection (with scissors) and removal of skin approximately 10 mm × 10 mm (Figure 2B).
20. Immediately after incision, reduce the inhalational anaesthetic concentration to ∼1% for maintenance and confirm adequate depth of anaesthesia by testing the tail pinch reflex.
21. Irrigate the graft bed with sterile normal saline.
22. Place the graft in the bed (Figure 2C).
23. Secure the edges of the graft with small amounts of clinical grade cyanoacrylate tissue adhesive (Dermabond or Vetbond) (Figure 2D).
24. Remove the rectal probe.
25. Place a non-adhesive absorptive dressing on top of the graft and secure firmly with an adhesive cloth bandage (Figure 2E).
26. Cease inhalational anaesthesia and transfer the recipient mouse to the recovery cage.

**CRITICAL:** Only apply adhesive to the edges of the graft and ensure that the wound edges are closely apposed.

**CRITICAL:** Bandages must be applied tightly to prevent removal by mice. However, not so tightly that their breathing is restricted.

### Part 2: Recovery and monitoring

**Timing: Recovery – 30 min & monitoring – intermittently over 7 weeks**

This section describes the procedure for post-operative recovery and monitoring of skin grafted mice.

27. Immediately post-operatively, allow the recipient mouse to recover individually in an empty cage lined with a disposable towel preheated on a heat mat set to 38°C.
28. Place only half the recovery cage on the heating mat to ensure the recipient mouse has access to a cooler environment to prevent overheating.
29. Monitor the recipient mouse for a minimum of 15 min and once awake and exploring in the recovery cage, return the recipient mouse to a holding cage.
30. Singly-house the recipient mice.

**CRITICAL:** In prior experience, group-housed mice frequently removed skin grafts from each other’s backs during the healing-in process, resulting in attrition from the experiment and sometimes causing severe bleeding if newly- formed blood vessels were disrupted.

31. Monitor the recipient mice twice for the first 5 days post-operatively, paying special attention to the detection of urethral plugging.
32. Assess the recipient mice on posture, activity, gait, respiratory pattern, hydration, bodily condition, hair, surgical wound, other abnormal signs and body weight (weight only performed once daily).
33. Monitor the recipient mice daily for the next 5 post-operative days.
34. Remove the bandage on day 7 post-transplantation.
35. Monitor the grafts for rejection.

***Note:*** the skin grafts are deemed rejected when less than 20% of the graft remains. The tempo of rejection depends upon the degree of mismatch between donor and recipient. Rejection generally occurs between 9 and 22 days post-transplant. Graft rejection can progress rapidly from inflammation to necrosis (Figure 2F). Rejected grafts may eventually scab and fall off.

**CRITICAL:** Urethral plugs can obstruct the urethra and lead to urinary retention. Untreated urethral obstruction will ultimately lead to renal failure.

### Part 3: AAV inoculation

**Timing: 40 min for a single mouse or 90 min for a group of 6 mice**

This section describes the steps involved in vector preparation for intravenous administration and injection of recipient mice.

#### Vector preparation

**Timing: 20 min**

36. Make single-dose aliquots from the stock solution in sterile 1.5 mL microcentrifuge tubes and store them at –80°C.
  a. A single dose for each mouse is 5 × 10^11^ vgc.
  b. Before injection, thaw an aliquot on ice and add 500 μL of sterile PBS into the microcentrifuge tube.
  c. Keep the diluted dose on ice and draw just before injection.

**CRITICAL:** All AAV work must be carried out in a biosafety cabinet with adequate personal protection.

#### Intravenous injection

**Timing: 20 min per mouse**

37. 30 days post-graft rejection, place the primed mouse in an induction chamber inside a biosafety cabinet.
38. Induce anaesthesia with isoflurane at 3% and supplemental oxygen at 0.6 L/min.
39. [male mice] Gently express the penis then stabilize it between the tips of blunt forceps. Visualise the penile vein and prepare the skin overlying the vein using chlorhexidine 0.5% in 70% ethanol.
40. [male mice] Administer the AAV vector slowly intravenously via the penile vein using a 30G needle.

***Note:*** the penile vein is larger than the tail vein and penile vein injection does not require pre-heating of the mice to dilate the blood vessels.

41. [male mice] After injection, retract the penis back into the abdomen and apply gentle pressure with a sterile cotton bud to achieve haemostasis.
42. [female mice] Warm the mouse using a heat lamp for 2–3 min and prepare the skin overlying the vein using chlorhexidine 0.5% in 70% ethanol.
43. [female mice] Administer the AAV vector slowly intravenously via the tail vein using a 30G needle.
44. [female mice] After injection, apply gentle pressure with a sterile cotton bud to the tail vein injection site to achieve haemostasis.
45. Immediately post-operatively, allow mice to recover individually in an empty cage lined with a disposable towel preheated on a heat mat set to 38°C.

***Note:*** Place only half the recovery cage on the heating mat to ensure the mouse has access to a cooler environment to prevent overheating.

46. Once the mouse has recovered, return it to its cage.

### Part 4: Peptide and tetramer preparation

**Timing: 5 h 15 min for MBL tetramers or 24 h 15 min for BioLegend Flex T tetramers**

This section describes how to prepare peptide-exchanged tetramers for alloreactive T cell staining.

#### Reconstitution of peptides

**Timing: 15 min**

47. Peptides were synthesized with an average of 98% purity by GL Biochem Shanghai Ltd.
  a. Dissolve lyophilized peptides (∼24 mg) individually in 500 μL of sterile DMSO.
  b. Dilute the dissolved peptides in sterile H_2_O to achieve a final concentration of 5 mM.
  c. Store the diluted solution at –80°C.

#### MBL tetramer preparation

**Timing: 5 h**

**Figure 3:**
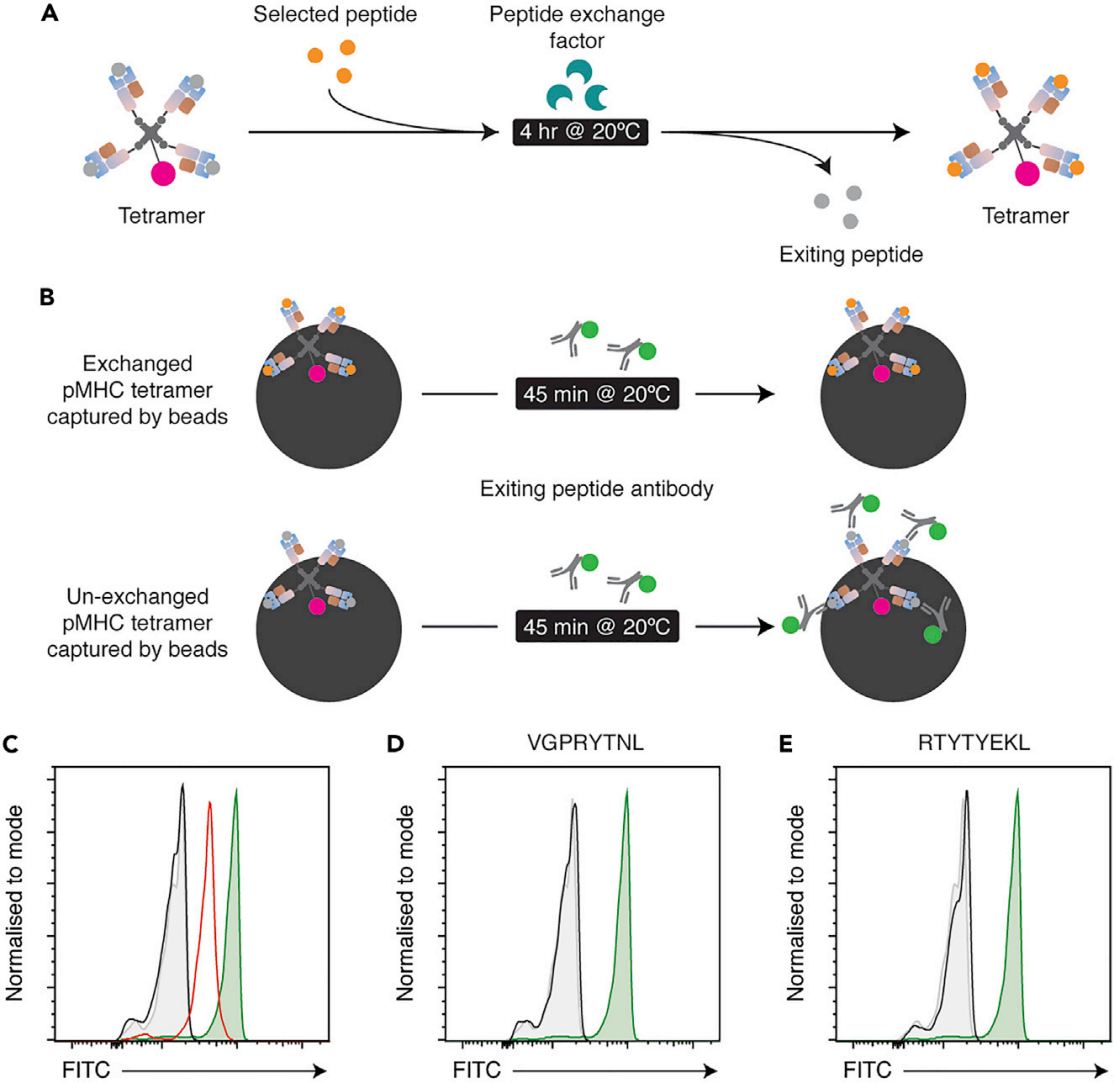
A schematic diagram of peptide exchange and quantitation of peptide exchange with the MBL QuickSwitch system (A) The MBL QuickSwitch system requires a peptide exchange factor to enzymatically exchange peptides. (B) Exchanged and un-exchanged tetramers can be captured by beads and subsequently analysed using flow cytometry. Using a FITC-conjugated antibody against the exiting peptide, we can detect un-exchanged tetramers. (C) The green histogram shows bead-immobilised un-exchanged tetramers stained with the FITC-conjugated antibody. The grey-filled histogram is a no-antibody control. To illustrate differences in exchange efficiency, we compared staining of tetramers made with either SNYLFTKL or AVVAFVMKM peptides. Tetramers exchanged with SNYLFTKL peptide show complete exchange efficiency (The exiting peptide is no longer present, black histogram is superimposed upon the no-antibody control – 99.5% efficiency). Tetramers exchanged with AVVAFVMKM show incomplete exchange (red histogram – 65.9% efficiency). (D and E) Tetramers exchanged with either VGPRYTNL or RTYTYEKL (black histograms) both show complete exchange efficiency. An alternative method of producing multimers should be used to avoid spurious results if exchange efficiency is less than 80%.

48. MBL QuickSwitch H-2K^b^ tetramers incorporating the selected peptides were generated and quantified according to the manufacturer’s protocol (Figure 3) [Link: https://www.mblintl.com/products/wp-content/uploads/sites/2/2021/02/15.17.1.8-Quickswitch-Quant-H-2-kb-ve3_rev1909_APedit.pdf].
  a. Pipette 50 μL of MBL QuickSwitch tetramer into a microtube.
  b. Add 1 μL of peptide and 1 μL of Peptide Exchange Factor sequentially, with gentle pipette mixing between each.
  c. Incubate the mixture at 20°C for 4 h protected from light.
  d. To quantitate peptide exchange, tetramers are captured by beads and then incubated with a FITC conjugated antibody against the exiting peptide.

#### BioLegend Flex-T multimer preparation

**Timing: 24 h**

**Figure 4:**
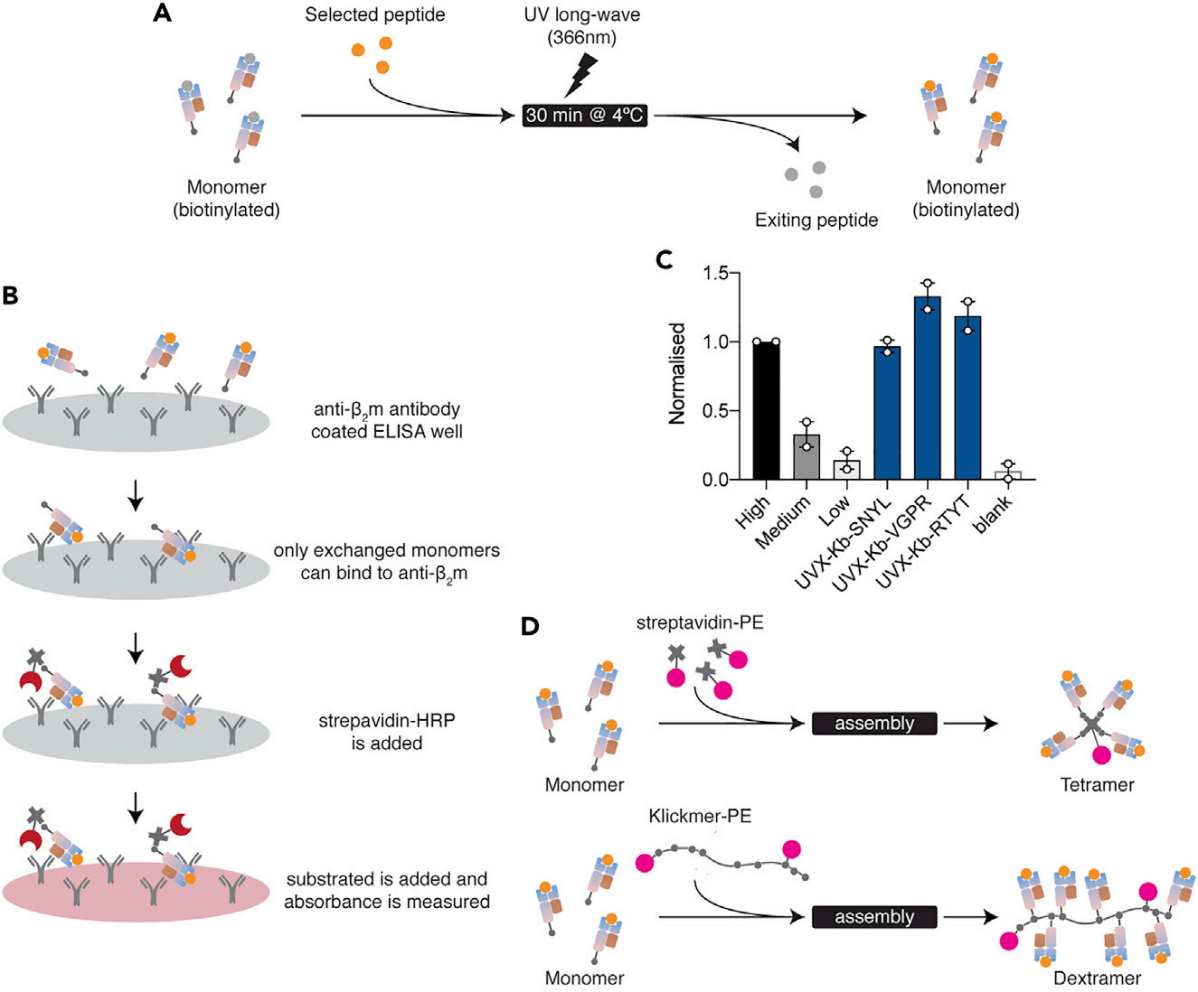
A schematic diagram of peptide exchange and quantitation of peptide exchange with the BioLegend Flex-T system (A) BioLegend Flex-T system monomers exchange their peptides through a UV-mediated process (long-wave 366 nm). (B and C) The peptide exchange efficiency can be quantified using the associated ELISA kit. Three peptides are shown in this graph (SNYLFTKL, VGPRYTNL, RTYTYEKL). The absorbance reading for the “high” standard was arbitrarily assigned a value of 1.0 and other readings were normalised to this as recommended by the manufacturer. Bars represent the mean of duplicate readings, the individual values are shown as dots. Monomers for further use should show an absorbance value at least as high as that for the “medium” standard. (D) UVX monomers can be assembled with either PE-conjugated streptavidin or PE-conjugated Klickmer to form tetramers or dextramers, respectively.

49. BioLegend Flex-T H-2K^b^ tetramers incorporating the selected peptides were generated and quantified according to the manufacturer’s protocol (Figure 4) [Link 1: https://www.biolegend.com/en-us/protocols/flex-t-fixed-peptide-tetramer-preparation-and-flow-cytometry-staining-protocol. Link 2: https://www.biolegend.com/en-us/protocols/flex-t-hla-class-i-elisa-protocol].
  a. Pipette 20 μL of diluted peptide (400 μM) and 20 μL Flex-T monomer into a microtube.
  b. Illuminate the solution with long-wave 366 nm UV light for 30 min on ice, then incubate for 30 min at 37°C in the dark.
  c. Detect exchanged monomers (H-2 or HLA) using the LEGEND MAX™ Flex-T™ Human Class I Peptide Exchange ELISA Kit.
  d. [Tetramer generation] Transfer 30 μL of exchanged monomer into a microtube and add 3.3 μL of PE Streptavidin (0.2 mg/mL).
  e. [Tetramer generation] Incubate at 4°C for 30 min protected from light.
  f. [Tetramer generation] Prepare a blocking solution by mixing 1.6 μL of 50 mM D-Biotin and 6 μL of 10% (w/v) sodium azide in 192.4 μL of PBS.
  g. [Tetramer generation] Add 2.4 μL of blocking solution to the microtube containing the exchanged monomer and PE Streptavidin.
  h. [Tetramer generation] Incubate at 4°C for at least 16 h protected from light. The assembled tetramer is ready to be used.
  i. [Dextramer generation] Transfer 6 μL of exchanged monomer into a microtube and add 10 μL of 160 nM Klickmer-PE.
  j. [Dextramer generation] Incubate at 20°C for 30 min and store at 4°C.
  k. [Dextramer] The assembled dextramer is ready to be used.

**CRITICAL:** DMSO must be handled with adequate personal protection.

### Part 5: Day 7 liver leukocyte isolation

**Timing: 3 h**

This section describes the process of isolating enriched alloreactive T cells from the mouse liver.

50. 7 days post inoculation, place the mouse in an induction chamber and induce anaesthesia using isoflurane at 3% and supplemental oxygen at 0.6 L/min.
51. Perform midline laparotomy and expose the inferior vena cava by gently moving the intraperitoneal contents to the right.
52. Introduce a 22G cannula distal to the renal bifurcation and transect the hepatic portal vein to allow outflow of perfusate (Figure 5).

**Figure 5:**
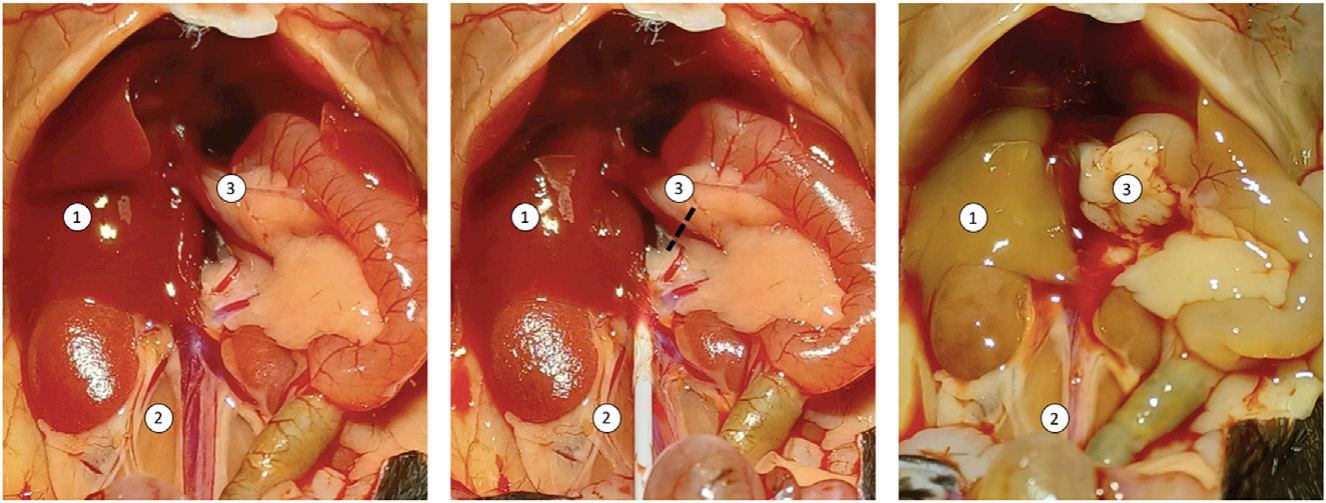
Liver retrograde perfusion Representative macroscopic images demonstrating the process of liver retrograde perfusion. 1: liver, 2: inferior vena cava, 3: portal vein. From left to right, the visceral anatomy was exposed, a cannula was introduced to the inferior vena cava, the liver turned pale after perfusion.
53. Slowly perfuse the liver with 20 mL of PBS at 20°C by manual injection over 1 min. Retrograde perfusion of the liver is achieved.
54. Transect the liver lobes and transfer them to a 100 μm cell strainer.
55. Gently press through and wash through with cold RPMI + 5% FCS (RPMI/FCS5) and then transfer the liver slurry into a 50 mL conical tube.
56. Wash the liver slurry twice with 50 mL RPMI/FCS5 by centrifugation at 400 × *g* for 10 min.
57. Decant the supernatant and resuspend the cell pellet in 15 mL PBS.
58. Add 9 mL of Isotonic Percoll PLUS and mix.
59. Centrifuge the solution at 500 × *g* for 15 min, with no brake.
60. Discard the supernatant (containing hepatocyte debris). The cell pellet contains the liver leukocytes.
61. Wash the liver leukocytes in the pellet once by centrifugation at 400 × *g* for 10 min.
62. Resuspend in 2 mL of red cell lysis solution.
63. Incubate for 2 min at 20°C.
64. Following this, wash the liver leukocytes twice by centrifugation at 400 × *g* for 10 min with RPMI/FCS5 as above, and count viable cells.

### Part 6: Tetramer and antibody staining

**Timing: 4 h 25 min**

This section describes the method for staining alloreactive T cells using peptide-exchanged tetramers and an antibody panel to determine recognition of pMHC epitopes.

#### Dasatinib preparation

**Timing: 10 min (for step 65)**

Dasatinib is a protein kinase inhibitor which was used in our protocol to enhance pMHC-tetramer staining. Dasatinib prevents T cell receptor (TCR) internalisation thereby increasing the density of cell-surface TCR available for tetramer binding.^8^^,^^9^

65. Dissolve dasatinib in sterile DMSO to 10 mM concentration and store aliquots at –80°C for no longer than 3 months. The working concentration of dasatinib for incubation with T cells is 50 nM.
  a. On the day of tetramer staining, thaw a dasatinib aliquot at 20°C.
  b. Dilute 10 μL of the 10 mM dasatinib solution in 2 mL of PBS. 1:200 dilution of the stock solution to achieve a concentration of 50,000 nM.
  c. Dilute 10 μL of the 50,000 nM dasatinib solution in 2 mL of PBS. 1:40,000 dilution of the stock solution to achieve a concentration of 250 nM. This will be used at step 68.

**CRITICAL:** DMSO must be handled with adequate personal protection.

#### Antibody preparation

**Timing: 15 min (for step 66)**

Fluorophore conjugated antibodies are used in conjunction with tetramer staining to identify surface markers. The list of antibodies is given in the key resources table.

66. Prepare the antibody panel in cold staining buffer (2% FCS in PBS) on the day of tetramer staining.

**CRITICAL:** CD8 antibody must be from the clone KT-15. Other clones can interfere with pMHC tetramer binding and reduce the efficiency of the staining.

#### Tetramer and antibody staining

**Timing: 4 h**

67. Wash the cells once with staining buffer by centrifugation at 400 × *g* for 10 min.
68. For each sample, resuspend 1 million cells in 40 μL of staining buffer.
69. Incubate the cells with 50 nM dasatinib for 30 min at 20°C by adding 10 μL of 250 nM dasatinib.
70. During the incubation period, spin the pMHC tetramer at 16,000 × *g* for 1 min to remove aggregates.
71. Add 0.5 μg of pMHC-PE tetramer to the cells incubate for 30 min at 20°C.
72. Following pMHC multimer staining, wash the cells twice with staining buffer by centrifugation at 400 × *g* for 10 min.
73. Incubate the cells with mouse TruStain FcX (BioLegend) (1 μL in 50 μL of staining buffer) for 10 min at 4°C.
74. Add 0.5 μg of purified monoclonal anti-PE (PE001, 1 μL in 50 μL of staining buffer) to the cells and incubate for 30 min on ice. Anti-PE antibody stabilises the multimers bound by their cognate TCRs by cross-linking these protein complexes.^9^
75. Wash the cells twice with staining buffer by centrifugation at 400 × *g* for 10 min.
76. Add surface antibodies (see key resources table - total volume of 100 μL in staining buffer per sample) to the cells and incubate for 30 min on ice.
77. Wash the cells twice with PBS by centrifugation at 400 × *g* for 10 min.
78. Add viability dye ZNIR (total volume of 100 μL in PBS per sample) and incubate on ice for 15 min.
79. Wash the cells twice with staining buffer by centrifugation at 400 × *g* for 10 min.
80. Resuspend the cells in 150 μL of staining buffer and filter through a 50 μm nylon mesh.
81. Cells are ready to be analysed using flow cytometry.

**CRITICAL:** We recommend including an FMO (no multimer) control as well as unstained and viability only controls. PD-1 negative bystander cells serve as an internal control population without T cells that respond specifically to the allogeneic pMHC.

## Expected outcomes

**Figure 6:**
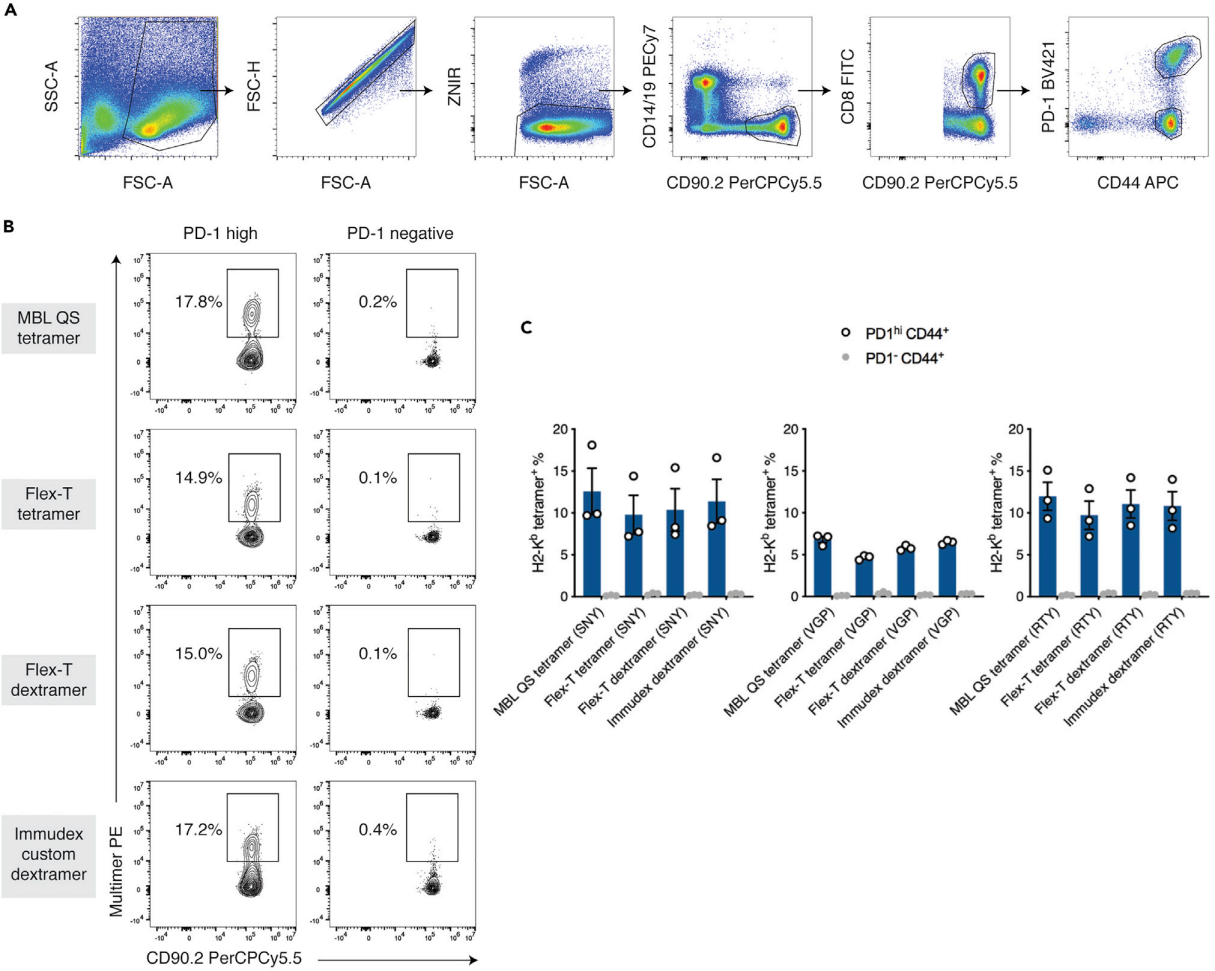
Identification of pMHC epitopes recognised by alloreactive CD8^+^ T cells and comparison of different pMHC-multimer assembly technologies (A) Gating strategy for identifying CD8^+^ T cells and stratifying them into two groups: CD44^+^PD-1^hi^ – activated alloreactive population and CD44^+^PD-1^–^ internal control population. (B) Representative images of flow cytometry results of different pMHC-multimer assembly technologies. (C) We tested four compositions - UVX monomers made into tetramers using strep-PE or dextramers with PE-Klickmer, against MBL quick-switch tetramer and a custom-produced dextramer from Immudex. The percentage of positive cells detected in the activated cell population was high and comparable across all reagents, while few multimer-positive cells were detected within the internal control population. Mean, SEM, and individual values are shown. n = 3 biological replicates.

To identify immunogenic peptides from the selected subset, we generated an enriched population of alloreactive CD8^+^ T cells by priming mice with a skin graft expressing H-2K^b^, followed by boosting with a liver-specific AAV vector encoding the H2-K^b^ heavy chain. From each mouse, we were able to obtain 3 to 8 million liver leukocytes. Activated CD8^+^ T cells were defined as CD44^+^PD-1^hi^, whereas CD44^+^PD-1^–^ cells constituted an internal control population which had been exposed *in vivo* to H-2K^b^ expressed on hepatocytes but was not activated. (Figure 6A). Peptides were deemed immunogenic when at least 2% of CD44^+^PD-1^hi^ CD8^+^ T cells were bound by pMHC tetramer. We were able to identify peptides that were recognised by a large percentage of the activated cell population while not being recognised by the internal control population.^1^

We compared the staining results for three highly immunogenic peptides using two commercially available peptide-exchange kits alongside custom-produced dextramers (Immudex, Denmark). Four compositions were tested – MBL quick-switch tetramer, Flex-T monomer (BioLegend, San Diego, CA) made into tetramers using strep-PE or assembled as dextramers with PE-Klickmer (Immudex, Denmark). The percentage of positive cells detected in the activated PD-1 high cell population was high across all tested conditions while producing clean staining with the PD-1 negative cell population. The proportion of activated alloreactive T cells detected using each method was comparable (Figures 6B and 6C).

### Downstream applications

Further applications of this protocol include tracking, immunophenotyping, TCR sequencing and gene expression analysis of alloreactive T cells. This pMHC multimer staining protocol has been optimised to include the use of a protein kinase inhibitor (dasatinib). Alloreactive T cells typically have low-moderate binding affinity for pMHC epitopes; accordingly, we found the use of a protein kinase inhibitor to be crucial in ensuring detection of the multimer-positive population.

**Figure 7:**
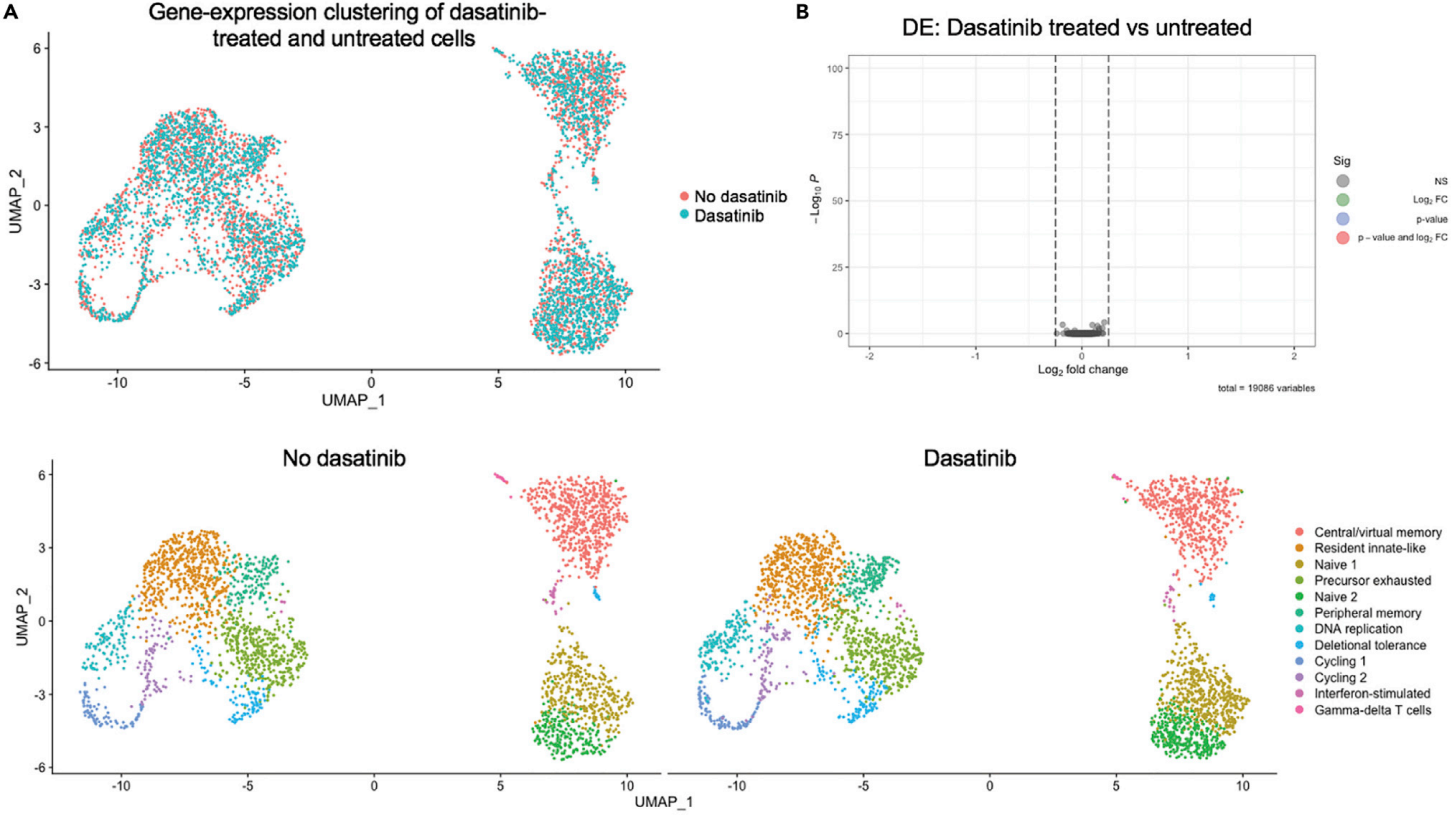
No transcriptomic changes resulted from brief treatment with dasatinib (A) scRNA-seq data were generated from liver leukocytes either treated or not treated with dasatinib. (A and B) (A) UMAP plots and (B) Volcano plots were generated. No differences in gene expression between the dasatinib-treated and untreated cells were detected.

Protein kinase inhibitors may also affect other signalling pathways, potentially confounding the downstream analysis of cells that have been incubated with dasatinib. To assess this, we treated isolated liver leukocytes with or without dasatinib, sorted them into activated, bystander and true naïve populations based on expression of CD44 and PD-1, and captured single cells using the BD Rhapsody system (BD Biosciences). Whole transcriptome libraries were prepared following the manufacturer’s instructions (BD catalogue 633801), sequenced on an Illumina NextSeq 2000 (Illumina, US) and analysed using the BD Rhapsody™ WTA Analysis Pipeline hosted on Seven Bridges Genomics, and subsequently with the Seurat Package in RStudio.^10^ No significant differences between the dasatinib-treated and untreated cells were observed, implying that brief treatment with dasatinib at ambient temperature does not substantially alter gene expression by CD8^+^ T cells (Figure 7).

## Limitations

Peptide exchange is most efficient for peptides with high affinity for the relevant MHC allomorph. Spurious results may arise if replacement of the exiting peptides is incomplete. Quantification of peptide exchange is necessary to exclude poorly-exchanged reagents, and confining the use of peptide-exchange methods to peptides with a predicted IC_50_ < 200 nM is recommended.^11^ The standard method for multimer production based on refolding of MHC class I monomers with the peptide of interest can be used for lower-affinity peptides.

Improper gating of multimer-positive cells can lead to misleading results. Several different types of control should be incorporated in the experimental design. We recommend including an FMO (no multimer) control. For alloreactive populations, it is not always possible to identify a negative-control multimer (comprising the allogeneic MHC with a non-immunogenic peptide) prior to the start of experimental work. A multimer of self-MHC with an abundant self-peptide can be useful here, but some level of TCR recognition may occur if the chosen self pMHC is responsible for positive thymic selection. Comparison of multimer-staining of activated CD8^+^ T cells with that of cell populations expected to have a much lower frequency of multimer-positive cells (e.g., bystander CD8+ T cells or CD4^+^ T cells) is very helpful, though it should be noted that strong TCR-pMHC binding does not always result in T cell activation.^12^

The pMHC multimer staining protocol has been developed including the use of a protein kinase inhibitor (dasatinib). As a result, concurrent intracellular staining for cytokines or other intracellular markers may not be possible. Most intracellular staining protocols require the use of Brefeldin A or Monensin to block protein transport and allow intracellular accumulation of cytokines.^13^ This step is not compatible with the pMHC multimer protocol.

## Troubleshooting

### Problem 1

Poor post-operative recovery (Part 1–2: recipient skin transplantation, recovery and monitoring).

### Potential solution

The procedure should ideally take less than 10 min per mouse. Only a small amount of chlorhexidine 0.5% in alcohol solution should be applied to the skin of the recipient mice (step 16). Too liberal application of alcohol solution can lead to significant heat loss and hypothermia in mice, contributing to poor recovery. Appropriate analgesia should also be used to help the recovery process (step 14).

### Problem 2

Skin graft failure post-operatively (Part 1–2: recipient skin transplantation, recovery and monitoring).

### Potential solution

We found from our experience that skin grafts can occasionally fail post-operatively. One cause for this is removal by the mouse (due to irritation) before the graft has properly healed into its bed. This usually occurs between day 1 and day 7 post-transplantation. Take care to apply the bandage securely (step 25). A new bandage must be applied if the mouse has removed it prior to healing-in. Poor healing due to poor surgical technique could also contribute to this problem. Avoiding the application of excessive tissue adhesive (step 23) is important to promote good healing.

### Problem 3

Failure to dissolve peptides (part 4: peptide and tetramer preparation).

### Potential solution

Finding the most appropriate solution for dissolving peptides can be challenging (step 47). Ideally, a solubility test should be carried out to determine the most ideal solution.

### Problem 4

Variability in peptide exchange efficiency can influence staining results (part 4: peptide and tetramer preparation).

### Potential solution

Only use exchanged tetramers where the extent of exchange reaches a threshold of 80% (MBL QuickSwitch) or equivalent to the “medium” standard (BioLegend Flex-T) (steps 48 and 49). We recommend using peptides with a predicted IC_50_ less than 200 nM for exchange. If screening of lower affinity peptides is desired, consider using traditional refolded tetramers.

### Problem 5

Poor liver perfusion (part 5: day 7 liver leukocyte isolation).

### Potential solution

During perfusion with PBS, the colour of the liver should change quickly from red to pale (step 53). If this does not occur, the liver is not perfused adequately. Ensure that cannula is in the IVC (step 52). Also ensure that the portal vein is completely transected (step 53).

### Problem 6

A low number of liver leukocytes are isolated (part 3: AAV inoculation, part 5: day 7 liver leukocyte isolation).

### Potential solution

There are various possible causes. Firstly, the recipient mice may not have mounted an effective immune response against the allograft. This is more likely if the graft has been compromised for technical reasons. Such mice are not properly primed. Poor intravenous injection technique can reduce delivery of the vector to hepatocytes (steps 37–46). Robust expression of the allogeneic MHC-class I transgene should be demonstrable in the liver.^1^^,^^3^ Problems with the liver leukocyte isolation prep (steps 55–64) can also lead to a lower number of liver leukocytes being isolated. Be certain that the centrifuge has come up to speed before leaving it, and make sure that the brake is off. Check for a visible pellet before removing the supernatant.

## Resource availability

### Lead contact

Further information and requests for resources and reagents should be directed to and will be fulfilled by the lead contact, Alexandra Sharland (alexandra.sharland@sydney.edu.au).

### Materials availability

The AAV2/8 vector was packaged, purified, and quantitated by the Vector and Genome Engineering Facility, Children’s Medical Research Institute, Westmead, 2145, and Australia. Peptides were synthesized with an average of 98% purity by GL Biochem Shanghai Ltd.

## Data Availability

Code for the analysis of dasatinib effect upon the transcriptome of alloreactive, bystander or naive CD8^+^ T cells is available from the authors upon reasonable request. Original data have been deposited to the Gene Expression Omnibus (NCBI GEO: GSE217149).
